# Role of TGF-Beta and Smad7 in Gut Inflammation, Fibrosis and Cancer

**DOI:** 10.3390/biom11010017

**Published:** 2020-12-27

**Authors:** Carmine Stolfi, Edoardo Troncone, Irene Marafini, Giovanni Monteleone

**Affiliations:** 1 Department of Systems Medicine, University of Rome “Tor Vergata”, 00133 Rome, Italy; carmine.stolfi@uniroma2.it (C.S.); troncone.edoardo@gmail.com (E.T.); irene.marafini@gmail.com (I.M.); 2 Division of Clinical Biochemistry and Clinical Molecular Biology, University of Rome “Tor Vergata”, 00133 Rome, Italy

**Keywords:** colorectal cancer, gastric cancer, cytokines, inflammatory bowel diseases, Crohn’s disease, epithelial-mesenchymal transition, Th17 cells, TNF-α, antisense oligonucleotides, mucosal immunity

## Abstract

The human gastrointestinal tract contains the largest population of immune cells in the body and this is a reflection of the fact that it is continuously exposed to a myriad of dietary and bacterial antigens. Although these cells produce a variety of inflammatory cytokines that could potentially promote tissue damage, in normal conditions the mucosal immune response is tightly controlled by counter-regulatory factors, which help induce and maintain gut homeostasis and tolerance. One such factor is transforming growth factor (TGF)-β1, a cytokine produced by multiple lineages of leukocytes, stromal cells and epithelial cells, and virtually targets all the gut mucosal cell types. Indeed, studies in animals and humans have shown that defects in TGF-β1 production and/or signaling can lead to the development of immune-inflammatory pathologies, fibrosis and cancer in the gut. Here, we review and discuss the available evidence about the role of TGF-β1 and Smad7, an inhibitor of TGF-β1 activity, in gut inflammation, fibrosis and cancer with particular regard to the contribution of these two molecules in the pathogenesis of inflammatory bowel diseases and colon cancer.

## 1. Introduction

The human gastrointestinal (GI) tract harbors the largest population of immune cells in the body and this is the result of a continuous exposure of the gut immune system to a complex and dynamic population of microorganisms (e.g., commensal bacterial, viral and fungal species) and dietary antigens [1]. In order to keep a state of “physiological inflammation”, which contributes to deal with invading pathogens, while preserving barrier integrity and allowing normal absorptive and digestive functions, many intestinal immune and non-immune cells produce a large amount of counter-regulatory biomolecules, which contribute to maintain gut homeostasis and tolerance [2]. Changes in the expression/function of such molecules contribute to initiate and/or propagate detrimental signals, which can eventually result in pathological conditions [2].

Among these molecules, a crucial enforcer is transforming growth factor (TGF)-β1, a cytokine produced by multiple lineages of leukocytes, stromal and epithelial cells and virtually targets all the gut mucosal cell types [3].

TGF-β1 is a member of the TGF-β superfamily, which includes also TGF-β2, TGF-β3, bone morphogenetic proteins and several growth and differentiation factors [4]. TGF-β1 biological functions are initiated by two transmembrane receptors with serine/threonine kinase activity, namely TGF-β1 type 1 receptor (TβR1) and TGF-β1 type 2 receptor (TβR2) [5]. Specifically, binding of TGF-β1 to TβR2 leads to auto-phosphorylation of the receptor and subsequent recruitment of TβR1, to form a transmembrane heterodimer. Next, the kinase activity of TβR2 determines the phosphorylation/activation of the regulatory domain of TβR1 which, in turn, propagates the signal to a family of intracellular signal mediators known as Smads [6]. In detail, the activated TβR1-TβR2 complex promotes the phosphorylation/activation of Smad2 and Smad3 and their subsequent heterodimerization. Once activated, the Smad2/3 complex binds to Smad4 and moves into the nucleus to regulate the transcription of specific target genes [5,7]. The TGF-β1/Smad signaling is a strictly regulated phenomenon, and a protein belonging to Smad proteins, namely Smad7, represents one of the main negative regulators of such a pathway, acting both in the nucleus and in the cytoplasm through various mechanisms. In detail, Smad7 binds to TβR1 and competes with Smad2/3 for the catalytic site of phosphorylation, thus preventing the phosphorylation of Smad2/3 [8,9]. In addition, Smad7 can promote dephosphorylation/inactivation of TβR1 by recruiting phosphatases to the site [10]. Smad7 can also boost ubiquitination and proteasome-mediated degradation of TβR1 in association with SMURF1/2, an E3 ubiquitin ligases [11,12]. Finally, at nuclear level, Smad7 can exert its inhibitory activity by interfering with the formation of functional receptor-activated Smad/Smad4 complexes as well as their binding to DNA [13].

Besides its inhibitory effect on TGF-β1 signaling, Smad7 can affect the expression and function of several molecules involved in the control of both fibrotic and carcinogenetic processes in a TGF-β1-independent manner. We here review and discuss the role of TGF-β1/Smad7 axis in gut inflammation, fibrosis and cancer.

## 2. TGF-β1 Signaling and Intestinal Homeostasis

**Figure 1:**
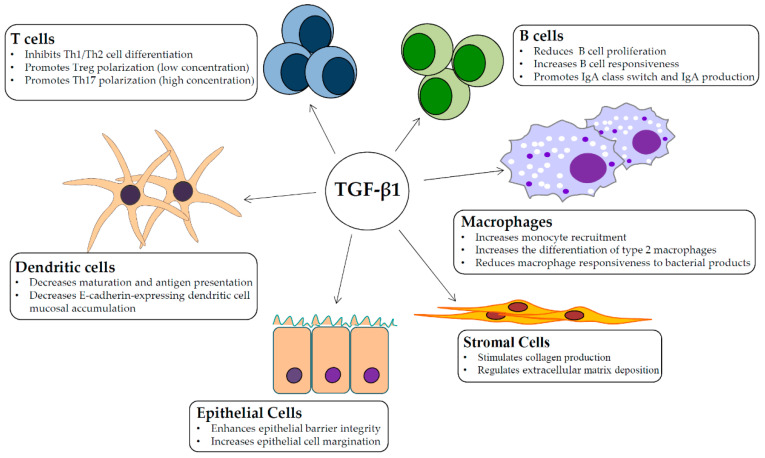
Schematic view of the main cell targets and biological function of transforming growth factor (TGF)-β1 in the gut.

The intestinal lamina propria (LP) is a loosely organized lymphoid compartment regarded as the major effector site for intestinal immune responses, with various kinds of immune cells communicating with one another through cell-cell contact and/or cytokine production [14]. The immune cell infiltrate within the LP is substantial, encompassing both innate immune cells, such as dendritic cells (DCs), macrophages and innate lymphoid cells (ILCs), and adoptive immune cells, such as T lymphocytes—of which the majority are CD4+ T helper (Th) and T regulatory (Treg) lymphocytes—plasma cells (mainly IgA+ and to a lesser extent IgG+ and IgM+), and B lymphocytes [14]. The function of many mucosal cell types is regulated by TGF-β1 signaling via autocrine and paracrine effects (Figure 1).

TGF-β1 markedly restrains both activation and proliferation of Th lymphocytes, thereby limiting effector functions that could lead to tissue destructive responses, such as those seen in autoimmune pathologies [15]. Consistently, genetically modified mice, which bear a dominant-negative TβR2 and are unresponsive to TGF-β1, as well as mice with T-cell targeted deletion of TβR2, develop systemic autoimmunity ultimately resulting in a severe colitis [16]. In both mouse strains, histopathology of tissues taken from multiple organs, including the gut, showed heavy lymphocyte infiltration and the presence of activated T cells, suggesting that the regulatory effects of TGF-β1 on T cells contribute to maintain intestinal immune homeostasis. Th lymphocytes present a high level of plasticity and are able to differentiate in various subsets depending on the specific cytokine milieu at induction and effector sites [17]. For instance, TGF-β1 markedly restrains the commitment of naïve T cells along the Th1 and Th2 lineages, which are important for responses against intracellular microbes and parasites, respectively, and whose aberrant activation in involved in the pathogenesis of inflammatory bowel diseases (IBD) [18]. In particular, the TGF-β1-induced inhibition of Th1 cell differentiation relies on the direct down-regulation of T-bet, a transcription factor described as the master regulator of Th1 cell commitment [19]. Moreover, TGF-β1 prevents the expansion of Th1 responses mediated by interleukin (IL)-12, the major driver of human Th1-type immune response [20], by reducing the expression of IL-12Rβ2 [21]. Concerning the TGF-β1-mediated inhibition of Th2 cell commitment, this relies on the direct down-regulation of GATA3, a transcription factor taking part in Th2 cell differentiation [22,23]. Notably, TGF-β1 can also promote the polarization of T cells towards a regulatory phenotype, namely T regulatory cells, both directly and indirectly [24]. Tregs, which are characterized by the expression of the transcription factor Forkhead box p3 (Foxp3) [25], play a key role in modulating the immune response, thereby maintaining homeostasis and self-tolerance, mainly acting on effector T cells [26]. Mice with non-functional TGF-β1 signaling caused either by TGF-β1 deficiency or T cell-specific deletion of TβRII showed a pronounced decrease in peripheral CD4+Foxp3+Tregs, suggesting that TGF-β1 may contribute to the gut homeostasis in part by triggering Treg differentiation [27,28]. This assumption is reinforced by evidence indicating that TGF-β1 promotes the formation of naturally occurring Tregs, a Treg subset present in the thymus early after birth, as well as of peripherally induced Tregs, which differentiate from naïve T cells in peripheral organs [29,30,31]. Of note, TGF-β1-deficient mice, but not mice with a targeted TGF-β1 deletion on CD4+ T cells, present a diminished fraction of CD4+Foxp3+Tregs [28], thus indicating that TGF-β1 released by other cell populations is pivotal in peripheral Treg differentiation. CD103-expressing DCs, a subset of DCs primarily involved in cross-presentation of self or foreign antigens, induction of gut-homing molecules on effector T cells as well as in the generation of Tregs [32], are a major source of TGF-β1 in the gut [33]. In addition, CD103+ DCs synthesize high levels of retinoic acid (RA), which potentiates TGF-β1-induced Treg expansion via a direct action on Foxp3 promoter [34]. Besides the above-mentioned effects, TGF-β1 and RA cooperate to the in vitro differentiation of naïve T cells into another subset of Foxp3-expressing Tregs, termed induced Tregs [33].

Concerning the cross-talk between gut microbiota and the intestinal immune system, TGF-β1 produced by colonic lamina propria DCs fosters the generation of Tregs following *Clostridium butyricum* infection [35]. Moreover, together with IL-6, IL-21 and IL-1β, TGF-β1 contributes to the differentiation of Th17 cells, a Th cell subset characterized by the expression of the master regulator retinoid acid-related orphan receptor (ROR)-γt and producing a wide range of cytokines, including IL-17A, IL-17F, IL-21 and IL-22 [36]. Of note, the commitment of naïve CD4+ T cells toward a Th17 or Treg phenotype has been seen to rely on TGF-β1 concentration [37]. Indeed, low TGF-β1 concentration induce Treg differentiation via the down-regulation of IL-23 receptor, whereas high concentration of TGF-β1, simultaneously with IL-6 and IL-21, up-regulates IL-23 receptor thus promoting Th17 polarization [38].

The tissue specificity of lymphocyte homing, a process that facilitates the access of such immune cells to specific tissues and organs, is tightly controlled by the interaction between the homing molecules (e.g., selectins, integrins) on lymphocytes and their specific ligands on the vascular endothelial cells of different tissues [39]. TGF-β1 dampens the gut homing capacity of effector CD8+ T cells from the secondary lymphoid organs to the intestine by inhibiting the expression of integrin α4β7. Consistently, transgenic mice bearing TβR2 deficient T cells showed decreased retention of antigen-specific memory CD8+ T cells in the intestinal tissues partly due to the defective expression of αEβ7 and α1 integrins, as well as CD69 [40].

TGF-β1 also plays a key role in the regulation of B cell and plasma cell biology. In B cells, TGF-β1 mediates IgA class switching and promotes IgA synthesis [41,42]. Although mice with non-functional TGF-β1 signaling in B cells do not develop intestinal inflammation, the deletion of TβR2 in CD19-expressing B cells associates with B cell hyperplasia in the isolated or aggregated lymphoid follicles forming Peyer’s patches and hampered B cell responsiveness resulting in a complete serum IgA deficiency [41,43]. Secretory IgAs are critical for the control of the intestinal microbiota. For instance, they can protect against luminal bacteria via direct neutralization and by enhancing DC phagocytosis and antigen presentation. In addition, secretory IgAs can impede the adhesion of bacteria to the epithelium by blocking their surface expressed epitopes [44]. In Peyer’s patches, IgA production increases following interaction between B cells and DCs via TGF-β1-activated integrin αvβ8 [45]. Of note, TGF-β1 controls IgA production via the canonical Smad-mediated cascade. In particular, Smad2 deficiency results in a lack of IgAs, whereas ectopic overexpression of Smad3 and Smad4 results in an augmented IgA production [46,47].

Innate lymphoid cells (ILCs) are a family of innate immune cells involved in immune homeostasis, tissue remodeling and host defense (contributing to the front line against pathogens) [48]. These cells mirror the phenotypes and functions of T cells and are abundantly present at mucosal sites [48]. Although there is no clear evidence that TGF-β1 controls the function of ILCs in the gut, recent studies indicate that TGF-β1 may regulate the differentiation/development of such cells in other districts [49,50].

TGF-β1 plays a pivotal role in modulating both expansion and function of intestinal DCs thus preventing immune defects that may result in inflammatory bowel diseases and autoimmunity. Indeed, mice bearing a specific TβR2 deletion in DCs spontaneously developed systemic autoimmunity and colitis, with the latter characterized by goblet cell depletion, marked mucosal lymphocytic infiltration, hampered expansion/functionality of Tregs, presence of activated T cells and B cells, and increased secretion of inflammatory cytokines [51,52]. In addition to TGF-β1 production, DCs contribute to TGF-β1 activation. Travis and colleagues elegantly showed that TGF-β1 activation by DCs is essential for preventing immune dysfunctions leading to pathologic conditions [53]. In particular, conditional loss of the TGF-β1-activating integrin αvβ8 on leukocytes caused severe colitis and age-related autoimmunity in mice. This phenotype was largely due to lack of αvβ8 on DCs, as mice lacking αvβ8 mainly on DCs developed immunological abnormalities identical to those seen in mice lacking αvβ8 on all leukocytes, whereas mice lacking αvβ8 on T cells alone were phenotypically normal. Mechanistically, DCs lacking αvβ8 failed to induce Tregs, an effect that relied on reduced TGF-β1 activity [53]. Interestingly, TGF-β1 can control the mucosal accumulation of specific inflammatory DC subtypes. In this regard, Siddiqui et al. demonstrated that monocyte-derived inflammatory DCs expressing E-cadherin, the receptor for CD103, promoted intestinal inflammation. In a T cell transfer model of colitis, E-cadherin-positive DCs accumulated in the inflamed mesenteric lymph nodes and colon, had high expression of toll-like receptors, and produced colitogenic cytokines (e.g., IL-6, IL-23), after activation. Importantly, the presence of TGF-β1 led to a marked downregulation of E-cadherin expression by bone marrow-derived DCs in vitro and limited the accumulation of E-cadherin-positive DCs in vivo [54]. TGF-β1 regulates also monocyte/macrophage function. For instance, intestinal epithelial cell-produced TGF-β1 can behave as a chemokine and promote the recruitment of blood monocytes to the intestinal mucosa [55]. In addition, TGF-β1 stimulates the differentiation of type 2 macrophages, an immune cell subset with anti-inflammatory properties, and diminishes the responsiveness of macrophages to bacterial products, which is crucial in the maintenance of intestinal homeostasis [56]. Mice with TβR2 deficiency in macrophages do not develop a spontaneous colonic inflammation [57]. However, such animals exhibit increased susceptibility to dextran sodium sulphate (DSS)-induced colitis as well as reduced IL-10 levels, further pinpointing the ability of the cytokine to promote counter-regulatory signals in macrophages [57].

Finally, it is worth underlining the ability of TGF-β1 to target non-immune cells, such as epithelial cells and stromal cells, which are known to produce high amounts of the cytokine. TGF-β1 fosters the expression of claudin-1, a tight junction protein, as well as adhesion molecules (e.g., E-cadherin, vinculin), with the ultimate result to reinforce the epithelial barrier integrity [58]. Along the same line is the capacity of the cytokine to potently induce the margination of intestinal epithelial cells, a phenomenon that speeds-up the wound healing [59].

Selective suppression of TGF-β1 signaling in mouse intestinal epithelium does not result in a macroscopic inflammation. However, mice with such a defect are more prone to DSS-colitis as compared with sham [60].

## 3. TGF-β1/Smad Signaling and Intestinal Fibrosis

Fibrosis is a wound-healing response to either acute or chronic cellular injury that is characterized by the accumulation of extracellular matrix (ECM) [61]. Several conditions are involved in the initiation and development of fibrotic diseases such as chronic inflammation, oxidative stress, shear stress, hypoxia, as well as specific stimuli (e.g., basic fibroblast growth factor, Wnt family growth factors) [61]. TGF-β1 is a key regulator of ECM deposition and plays an important role in physiological repair processes [62]. Indeed, TGF-β1 was found to increase the expression of the major ECM proteins, fibronectin and collagen, in cultured mesenchymal and epithelial cells [63]. In vivo, when injected subcutaneously in newborn mice, TGF-β1 induced collagen accumulation and a fibrotic tissue response at the site of injection [64]. Subsequent studies of fibrotic disease pathogenesis in several organs, such as liver, lung, kidney and skin, indicated that TGF-β1 as well as its intracellular mediators (i.e., Smad proteins) are among the main factors promoting tissue fibrosis [65,66,67]. Indeed, TGF-β1 signaling is considered the key fibrogenic pathway, and thus a valuable therapeutic target, in both liver and pulmonary fibrosis [68,69]. In the former disease, TGF-β1—produced by hepatic stellate cells (HSCs), immune (e.g., macrophages, platelets), and non-immune cells (e.g., hepatocytes)—triggers fibrosis by driving HSC activation and trans-differentiation to myofibroblasts, which are the main producers of collagen and other ECM proteins in the liver [68]. Interestingly, different functions have been attributed to the Smad proteins (e.g., Smad2, Smad3 and Smad7) in liver fibro-proliferative disorders depending on cell types [68]. In the lungs, TGF-β1 produced by a variety of cell types, such as alveolar macrophages, activated alveolar epithelial cells and fibroblasts, induces monocyte and fibroblast recruitment as well as fibroblast proliferation via platelet-derived growth factor (PDGF). In these cells, TGF-β1 also promotes the synthesis of inflammatory/fibrogenic cytokines, including PDGF, tumor necrosis factor (TNF)-α and IL-1β, further enhancing and perpetuating the fibrotic response [69]. Other pathways proposed as pathogenic mechanisms of lung fibrosis include TGF-β1 activation mediated by proteases, in particular secretory leukocyte protein [70,71]. For detailed information on the mechanisms underlying TGF-β1 signaling-mediated fibrogenesis in organs other than the gastrointestinal tract, we refer the reader to other more specific reviews [72,73,74].

As pointed-out above, TGF-β1 stimulates stromal cells to produce fibrogenic mediators and regulators of ECM deposition. In addition, TGF-β1 promotes the differentiation of mesenchymal cells in myofibroblasts, which display contractile activity and produce collagen and fibronectin, thereby facilitating wound repair [75,76]. For these reasons, TGF-β1 is considered as a major fibrogenic cytokine and a poorly controlled TGF-β1 activity has been involved in the development of intestinal fibrosis and strictures, which may complicate the natural history of Crohn’s disease (CD) [77]. Intestinal strictures in CD patients were associated with an increased TGF-β1 transcript level and excessive accumulation of extracellular matrix proteins, such as collagens and fibronectin [78,79]. Myofibroblasts isolated from intestinal strictures of CD patients overexpress collagen III, and TGF-β1 promotes collagen III production by myofibroblasts [79]. There is also evidence that anti-fibrogenic drugs used for the treatment of fibrotic diseases (i.e., pirfenidone) suppress intestinal fibrosis in a DSS-induced colitis model by inhibiting TGF-β signaling [80,81].

In line with the fibrogenic role of TGF-β1 in the human gut, TGF-β1 overexpression in the intestine of mice resulted in the development of intestinal fibrosis [82]. More recently, Flier et al. showed that TGF-β1-driven epithelial-mesenchymal transition (EMT) contributed to intestinal fibrosis in a rodent model of CD and that inhibition of TGF-β1 prevented this process as well as fibrosis [83].

Although the fibrogenic role of TGF-β1 in the gut is well accepted [62,78], it is worth underlining that additional factors/cytokines, which are highly produced in the inflamed tissue of CD patients (e.g., TNF–α), can stimulate stromal cells to synthesize elevated amounts of collagen, thus contributing to the pathogenesis of CD strictures [84,85]. In this context, defective TGF-β1 signaling, resulting in an impaired activity of the key transduction protein Smad3 and associated with elevated levels of the inhibitory protein Smad7, was seen in the mucosa of CD patients [86]. Knockdown of Smad7 with a specific antisense oligonucleotide (ASO) restored the ability of TGF-β1 to hamper the production of inflammatory cytokines in CD mucosal cells [86] and attenuated 2,4,6-trinitrobenzene sulfonic acid (TNBS)-driven experimental colitis (mimicking human CD) in mice [87]. Consistently, a phase 1 study showed that oral administration of a Smad7 ASO-containing drug, denominated GED-0301 and, later on, Mongersen, in patients with active, non-stricturing, non-perforating CD, was safe and associated with clinical benefit [88]. Subsequently, two independent phase 2 studies showed that Mongersen induced clinical and endoscopic improvement in steroid-dependent and/or resistant CD patients [89,90], although a phase three trial was discontinued in October 2017 due to an interim analysis that documented an apparent lack of efficacy of the drug [91]. Interestingly, in a follow-up study of the phase one trial of GED-0301, no strictures were observed, by small intestine contrast ultrasonography, in CD patients treated with the drug for up to six months [92]. Moreover, at day 180, no patients had a change in the serum levels of the tissue inhibitor of matrix metalloproteinases-1, basic fibroblast growth factor and YKL-40 [92], which have been proposed as serum biomarkers for intestinal fibrosis [93]. In line with these findings was the observation that Mongersen hampered the fibrogenic process in a mouse model of TNBS-mediated colitis-driven intestinal fibrosis [94].Taken together, these findings highlight the complexity of TGF-β1 signaling in modulating the pathologic processes that may lead to intestinal fibrosis. Given such a complexity and the ability of TGF-β1 and Smad proteins to modulate key processes involved in gut carcinogenesis (reported and discussed in the next chapter), therapeutic options aimed at targeting TGF-β1 signaling components to treat intestinal fibrosis should be carefully weighted up to avoid the risks of enhancing colorectal cancer (CRC) development.

## 4. Role of TGF-β1/Smad Signaling in Colorectal Cancer

Altered expression/function of TGF-β1 and/or Smad proteins is commonly observed in cancers [95]. Interestingly, TGF-β1/Smad signaling has dual roles in cancer progression [96,97]. Indeed, while TGF-β may induce cell cycle arrest and apoptosis in transformed cells during tumor initiation, in the later stages of tumor development TGF-β1 signaling has been shown to promote processes that cancer cells may exploit to their advantage, such as dysregulated cell proliferation, stem-like behavior, EMT and angiogenesis. Similarly, accumulation of mutations in TGF-β1 pathway components during tumor progression may contribute to convert TGF-β1 behave from tumor-suppressive to tumor-promoting [96,97]. Such heterogeneity makes the output of the TGF-β1 response in cancer dependent on the stage and context of the disease.

Colorectal cancer, defined as a cancer arising in the human colon and/or rectum, is the third most frequently diagnosed cancer (with more than 1.8 million cases) and the second in terms of mortality, accounting for almost 900,000 deaths worldwide [98]. While the 5-year survival rate of CRC patients with nonmetastatic disease is more than 70%, it dramatically decreases to less than 20% in the presence of metastatic disease [99]. CRC arises as sporadic disease in approximately 70% of cases, with multiple genetic and environmental factors (most of which are still unknown) involved in the pathogenesis [86]. Instead, in 2% of cases, CRC can complicate the natural history of patients with colonic inflammatory bowel diseases (colitis-associated cancer (CAC)) [100,101], mainly of those with long standing ulcerative colitis (UC), with a cumulative risk that that is related to the extension/duration of the disease as well as to the severity of inflammation [102,103]. TGF-β1 signaling plays both carcinogenic and anti-carcinogenic roles in CRC depending on the stage and type of disease, likely reflecting the complexity of TGF-β1-affected processes. Increased TGF-β1 expression was seen in CRC compared to benign adenoma and noncancerous tissue [104]. While TGF-β1 induced growth arrest in well differentiated to moderate differentiated, localized CRCs, this did not happen in more aggressive cancers and metastatic carcinoma cells even these cells responded to TGF-β1 treatment by increasing their proliferation [105,106]. High TGF-β1 levels were observed in primary tumor specimens as well as in plasma taken from CRC patients and were correlated with metastatic disease and poor prognosis [105,107]. In this regard, TGF-β1 was broadly detected in human CRC liver metastases [108] and circulating TGF-β1 was indicated as a predictor of metastatic disease in patients who underwent resection for CRC [109]. Notably, Tauriello et al. recently showed that TGF-β1 inhibition prevented CRC metastasis by unleashing a cytotoxic T-cell response against cancer cells [110]. Mutations of *TGF-β receptor* and/or *Smad* genes have been observed in nearly 50% of CRCs and supposed to play a key role in colon carcinogenesis [111,112]. Mutations in *TβRII*, abrogating TGF-β signaling, occur late in the adenoma to carcinoma sequence [113] and have been detected in about 30% of all CRCs [114], and in more than 80% of CRCs presenting microsatellite instability (MSI-H) [115,116]. As MSI-H tumors, *TβRII* mutations mainly occur in the right colon rather than in other parts of the large intestine [117]. In this context, de Miranda and colleagues showed that TGF-β signaling may still remain active in some CRCs with a high level of MSI-H despite *TβRII* frameshift mutations [118]. The exact mechanism/s by which TβRII mutations contribute to CRC development are still unknown. However, some studies have suggested that inactivation of TβRII may induce (along with KRAS mutations) intestinal neoplasms in mice in a β-catenin-independent pathway [119] and enhance the expression of vascular epithelial growth factor-A, thereby increasing the metastatic potential of CRC cells [120]. Although less frequent, mutations in TβRI have also been detected in CRC [121]. In particular, deletion of three alanine residues from a nine alanine stretch in the N-terminal region of TβRI (TβRI*6A) was associated with an increased risk of CRC [122]. Despite these results have not been confirmed [123], a recent paper highlighted a role for TβRI*6A in promoting the migration and invasion of CRC cells [124]. Mutations of the downstream components of the TGF-β1 signaling pathway can also modulate colon carcinogenesis. Smad4 mutations have been detected in 8.6% of sporadic CRCs and commonly in the later stages of the disease [125]. Indeed, Smad4 mutations or loss of expression of Smad4 occur in up to one third of metastatic CRCs and are associated with poor prognosis [125], in contrast with the notion of a metastatic role of TGF-β1 signaling. The reason for such an apparent discrepancy may rely on the fact that, other than playing a pivotal role in the canonical TGF-β1 cascade, Smad4 is a central component of other signaling pathways [126]. In this context, Voorneveld and co-workers demonstrated that loss of Smad4 altered bone morphogenetic protein (BMP) signaling to promote CRC metastasis via activation of Rho and Rho-associated protein kinase (Rock) [127]. More recently, by investigating the specific role of Smad4 in colitis-associated CRC, Means et al. reported a loss of Smad4 protein in 48% of samples taken from patients with UC-associated carcinomas [128]. The authors showed that mice with deletion of Smad4 in the intestinal epithelium presented macroscopic invasive adenocarcinomas of the distal colon and rectum following chronic DSS-induced experimental colitis. Interestingly, the histopathologic analysis of the tumors showed a strong similarity with those occurring in human CAC. Mechanistically, the carcinogenic effect of *Smad4* epithelial deletion resulted in a strong inflammatory signature caused by the increased expression of numerous chemokines—in particular C-C motif chemokine 20 (CCL20)—leading to an excessive recruitment of immune-inflammatory cells [128].

Smad2 mutations occur in approximately 3%–6% of CRCs, more frequently in the early-stage of disease [125,129]. Both the *Smad2* and *Smad4* genes are located on a region of the chromosome 18q which is commonly deleted in CRC owing to a loss of the long arm of chromosome 18 (loss of heterozygosity) [130]. Mutations in Smad3 were also identified at a similar frequency of the Smad2 mutations in sporadic CRCs [125]. In addition to the somatic mutations described above, germline mutations in Smad4 and other components of the TGF-β signaling, such as BMPR1A, have been documented in patients with juvenile polyposis syndrome [131,132], which can develop into CRC [133]. *Smad7* gene variations have been extensively investigated in CRC patients. Boulay and colleagues analyzed the presence of Smad7 variants in 264 CRC specimens and found that patients with Smad7 deletion had a favorable clinical outcome compared with patients with Smad7 amplification [134]. Genetic variants within Smad7 gene have been linked to CRC development in two genome-wide association studies (GWAS) [135,136]. In both studies, a highly significant association with CRC was found for two single nucleotide polymorphisms (SNPs) in Smad7 (i.e., rs4939827, rs12953717). The association of these SNPs with CRC was thereafter proved by two other GWAS [137,138]. In 2016, a large-scale meta-analysis confirmed that several SNPs in Smad7 were associated with the risk of developing CRC [139]. More recently, a low-frequency coding variant in Smad7 (i.e., rs3764482), was associated with the risk of CRC in a Chinese population [140]. Finally, Campbell and co-workers reported an association whereby the common *Smad7* variant rs4939827 and body mass index may jointly influence the risk of developing CRC in women [141].

To address the role of Smad7 in colon carcinogenesis, Halder and co-workers stably over-expressed Smad7 in a TGF-β-sensitive, well-differentiated, and non-tumorigenic colonic cell line (termed FET). Ectopic Smad7 in FET cells increased their resistance against apoptosis and favored anchorage-independent cell growth as well as colony formation via a mechanism dependent on suppression of TGF-β signaling. Smad7-overexpressing FET cells also presented increased tumorigenicity compared to control cells in a xenograft mouse model [142]. In a following study, the same group showed that injection of Smad7-overexpressing FET cells in the spleen of athymic nude mice promoted the formation of liver metastasis [143]. The pro-metastatic role of Smad7 was associated with augmented level of junctional proteins, such as E-cadherin, claudin-1 and claudin-4, at distant sites [143].

Our studies indicated a link between Smad7 expression in immune cells and CAC. Specifically, we detected a reduced number of Smad7-expressing CD4+T lymphocytes in the colonic mucosa of inflammatory bowel disease patients who developed CAC compared to patients with uncomplicated disease [144]. In line with this finding, transgenic mice over-expressing Smad7 in T cells (Smad7 Tg mice) developed a more severe colitis, marked by an abundant infiltrate of cytotoxic CD8+ T cells and natural killer T cells, compared to control mice. Smad7 Tg mice were largely protected from tumors compared to sham, thus highlighting the opposing role of Smad7 in the control of sporadic and colitis-associated CRC [144]. The negative effect on colon carcinogenesis of Smad7 over-expression in T cells seemed to rely on the action of interferon-γ, as genetic ablation of such a cytokine in Smad7 Tg mice abolished the protective action of Smad7 [144]. Consistently with these observations, Smad7 Tg mice were less susceptible to graft tumors, produced by the subcutaneous injection of syngeneic colon adenocarcinoma cells (i.e., MC38), compared to wild-type littermates [145]. However, consistently with the genetic studies mentioned above, Smad7 showed a carcinogenic role in sporadic CRC. Indeed, we detected high Smad7 levels in CRC cells and Smad7 abrogation by a specific ASO hampered CRC cell growth both in vitro and in experimental models [146]. These effects relied on the modulation of cell cycle-related proteins, ultimately resulting in S phase arrest and cell death [146]. Our following studies revealed that Smad7 knockdown activated the eukaryotic translation initiation factor 2 α (eIF2α), a transcription factor involved in the regulation of cell cycle machinery, in a protein kinase RNA-dependent fashion, leading to CRC cell death [147]. More recently, Wang et al. reported that nuclear reporter subfamily 2, group F and member 2 (NR2F2), a protein involved in the development of several cancers [148], inhibited Smad7 expression and induced a TGF-β-dependent EMT of CRC cells [149], further underlining the dual role of Smad7 in the early and late stages of CRC development [96]. Altogether, these data highlight the complex role of TGF-β1/Smad7 signaling in colon carcinogenesis.

## 5. Conclusions

The findings discussed in this article underline the crucial role of TGF-β1/Smad cascade in the maintenance of intestinal homeostasis and indicate that defective function of this signaling pathway, due to gain/loss of function defects in the extracellular/intracellular signal transduction components (e.g., TβR2, Smad7), contribute to trigger and/or amplify detrimental signals in the gut, which may ultimately lead to intestinal inflammation, fibrosis as well as cancer. TGF-β1 signaling may exert opposite actions on both fibrogenic and carcinogenic processes in the gastrointestinal tract, depending on the location (i.e., upper and lower gastrointestinal tract) and stage of disease (i.e., early versus advanced). These apparently contradictory functions are not surprising given the complexity of this pathway, characterized by the interaction of its components, in diverse cell types, with a vast array of functionally heterogeneous molecules that may be differently expressed during such pathogenic processes. To clarify the role of TGF-β1 signaling/components in specific pathogenic contexts is an exciting challenge of future studies that may pave the way for the development of strategies aimed at attenuating/halting the course of these diseases.

## Data Availability

Not applicable.
